# Supplementary material for: Comprehensive analysis of single cell and bulk data develops a promising prognostic signature for improving immunotherapy responses in ovarian cancer
Source: PLoS One. 2024 Feb 12;19(2):e0298125. doi: 10.1371/journal.pone.0298125 (PMC10861092; doi:10.1371/journal.pone.0298125)
Supplement: S1 Fig — (DOCX) [file pone.0298125.s001.docx]

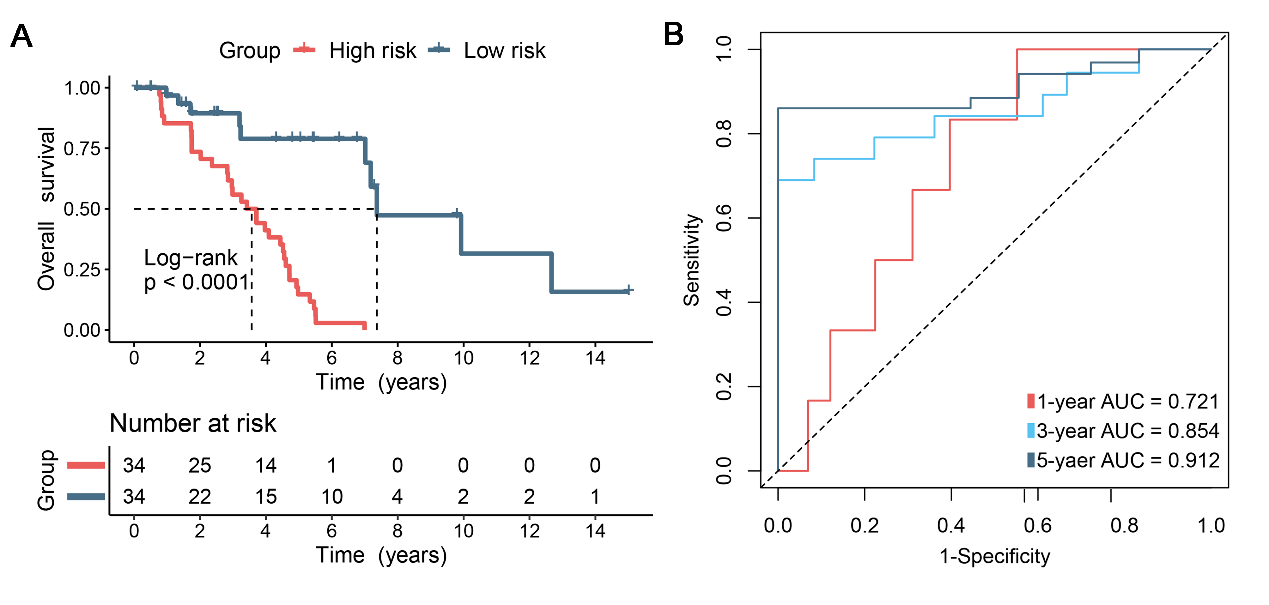


S1 Fig. The validation of prognosis signature based on in-house cohort. (A) Kaplan-Meier curves of overall survival (OS) according to the signature based on in-house cohort. (B) Time-dependent ROC analysis for predicting OS at 1/3/5 years based on in-house cohort.
