# Supplementary material for: Comprehensive analysis of single cell and bulk data develops a promising prognostic signature for improving immunotherapy responses in ovarian cancer
Source: PLoS One. 2024 Feb 12;19(2):e0298125. doi: 10.1371/journal.pone.0298125 (PMC10861092; doi:10.1371/journal.pone.0298125)
Supplement: S2 Fig — (DOCX) [file pone.0298125.s002.docx]

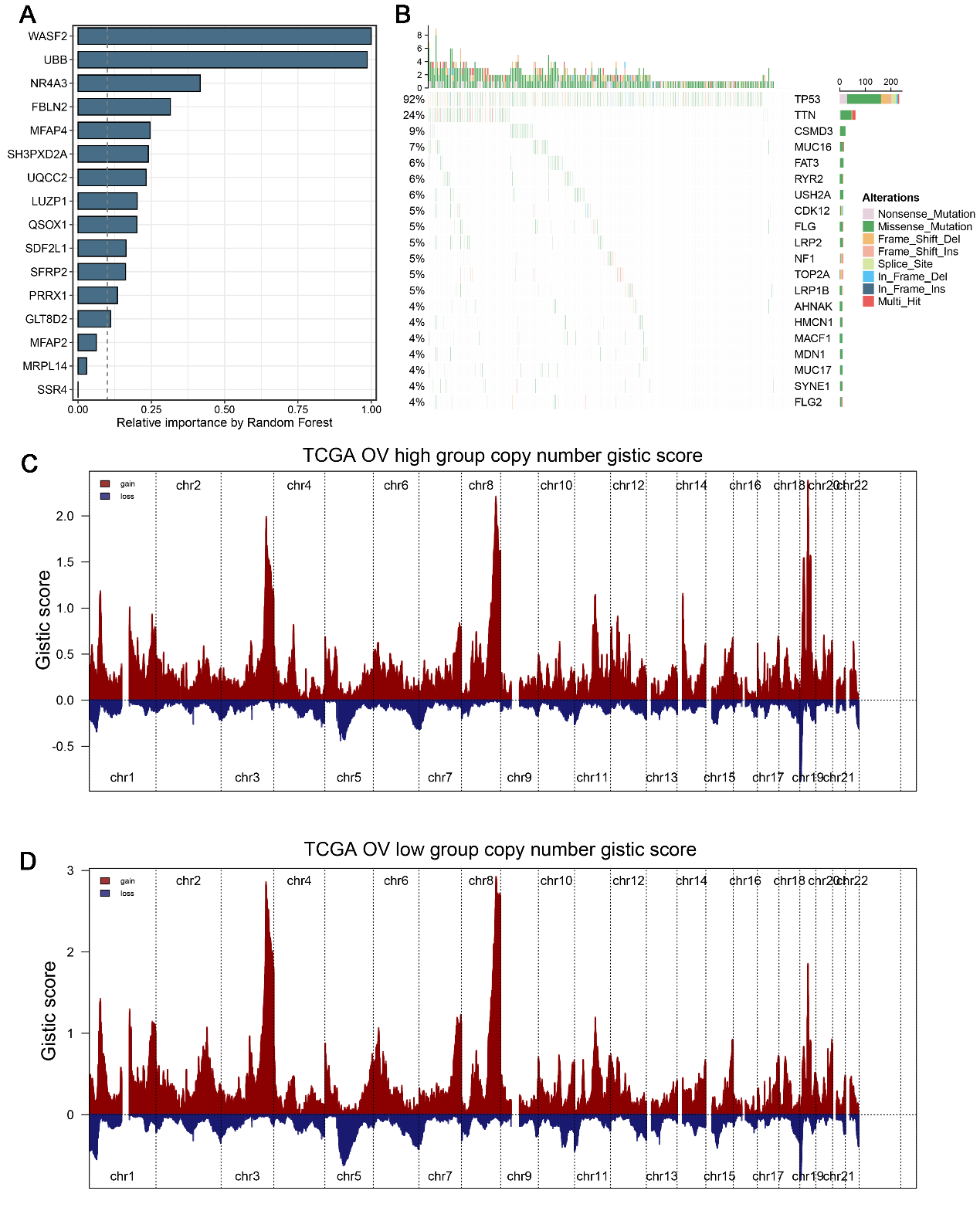


S2 Fig. The overall of somatic mutation and gistic score in ovarian cancer patients. (A) The importance values of candidate genes by random forest algorithm. (B) The landscape of somatic mutation with top 20 frequently mutated genes via waterfall plot. (C) The gain and loss of copy number gistic score in high-risk group. (D) The gain and loss of copy number gistic score in low-risk group.
