# Supplementary material for: Comprehensive analysis of single cell and bulk data develops a promising prognostic signature for improving immunotherapy responses in ovarian cancer
Source: PLoS One. 2024 Feb 12;19(2):e0298125. doi: 10.1371/journal.pone.0298125 (PMC10861092; doi:10.1371/journal.pone.0298125)
Supplement: S3 Fig — (DOCX) [file pone.0298125.s003.docx]

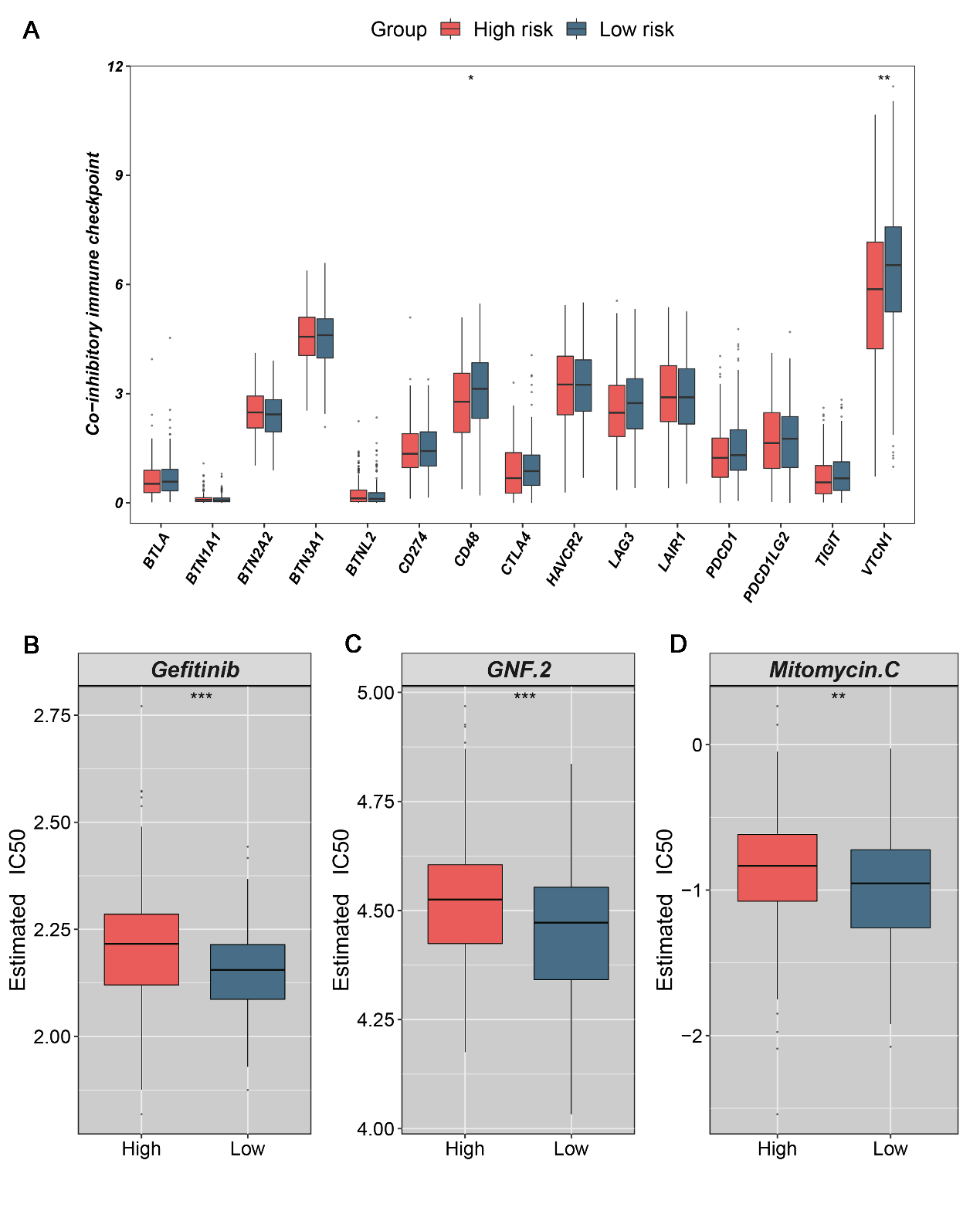


S3 Fig. The expression of immune checkpoints and the distribution of IC50 value. (A) Distribution of co-inhibitory molecules between high-risk and low-risk groups. (B-D) The identification of potential therapeutic drugs for patients in low-risk group, including Gefitinib (B), GNF.2 (C), and Mitomycin.C (D). *P< 0.05, **P< 0.01, ***P< 0.001.
