# Supplementary material for: Comprehensive analysis of single cell and bulk data develops a promising prognostic signature for improving immunotherapy responses in ovarian cancer
Source: PLoS One. 2024 Feb 12;19(2):e0298125. doi: 10.1371/journal.pone.0298125 (PMC10861092; doi:10.1371/journal.pone.0298125)
Supplement: S4 Fig — (DOCX) [file pone.0298125.s004.docx]

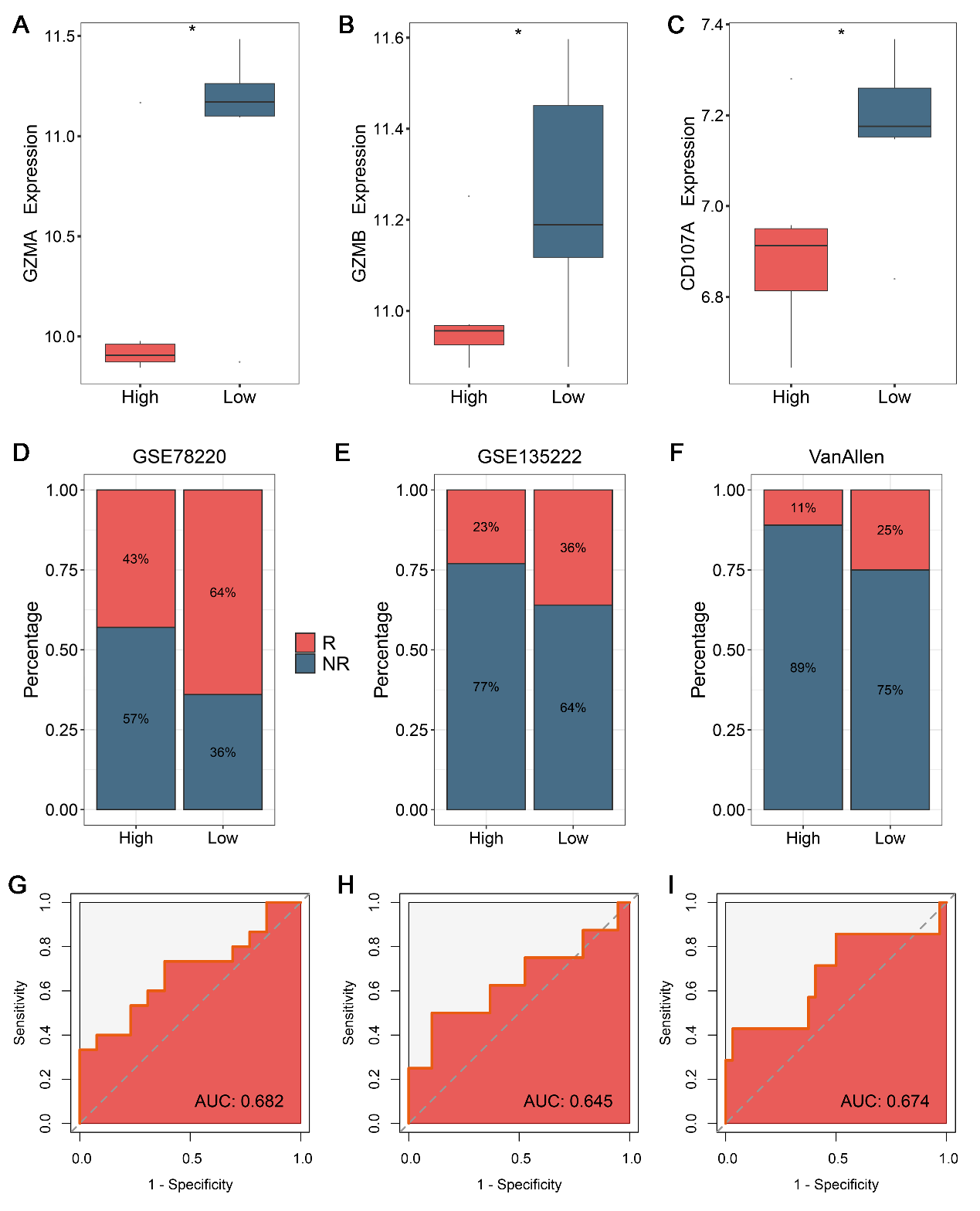


S4 Fig. The evaluation of the risk scoring system’s predictive performance in immunotherapy. (A-C) Distributions of GZMA expression (A), GZMB expression (B), and CD107A expression (C) between high-risk and low-risk groups in GSE160755. *P< 0.05. (D-F) Immunotherapy response ratio of between high-risk and low-risk groups in GSE78220 (D), GSE135222 (E), and VanAllen cohorts (F). (G-I) Receiver operating characteristic curves of the signature to predict the benefits of immunotherapy in GSE78220 (G), GSE135222 (H), and VanAllen cohorts (I).
