# Supplementary material for: Comprehensive analysis of single cell and bulk data develops a promising prognostic signature for improving immunotherapy responses in ovarian cancer
Source: PLoS One. 2024 Feb 12;19(2):e0298125. doi: 10.1371/journal.pone.0298125 (PMC10861092; doi:10.1371/journal.pone.0298125)
Supplement: S1 File — (DOCX) [file pone.0298125.s005.docx]

All the source codes for each figure are available in the following link: <https://www.jianguoyun.com/p/Ddy8K4sQsITxCxibypgFIAA>. The further inquiries can be directed to the first author and corresponding author.
